# ﻿Description of a new nematode species, *Chromadorinacommunis* sp. nov. (Nematoda, Chromadoridae), from Changdao Island, China and phylogenetic analysis of Chromadorida based on small subunit rRNA gene sequences

**DOI:** 10.3897/zookeys.1159.100908

**Published:** 2023-04-25

**Authors:** Wen Guo, Mengna Wang, Haotian Li, Chunming Wang

**Affiliations:** 1 College of Life Sciences, Liaocheng University, Liaocheng, 252059, China Liaocheng University Liaocheng China

**Keywords:** Chromadorida, free-living marine nematodes, phylogenetic analysis, SSU, taxonomy

## Abstract

*Chromadorinacommunis***sp. nov.** is described from Changdao Island at the confluence of the Yellow and the Bohai seas. The new species is characterized by its medium-sized body; finely striated cuticle with homogeneous punctations; absence of ocelli; buccal cavity with three equal-sized, solid teeth; four cephalic setae; oval amphidial fovea which is positioned between cephalic setae; curved spicules with tapered distal ends; simple, boat-shaped gubernaculums; five or six cup-shaped precloacal supplements; and conical tail with a very short spinneret. A phylogenetic analysis of small subunit rRNA gene sequences using maximum-likelihood and Bayesin inference confirmed the taxonomic position of *Chromadorinacommunis***sp. nov.** within Chromadorinae. Tree topology in Chromadorida shows six morphological families clustered into a monophyletic clade and verifies the taxonomic position of the family Neotonchidae based on morphological and molecular analysis.

## ﻿Introduction

Nematodes are one of the most widely distributed and diverse groups of the animal phyla, and half a million to 10 million nematode species have been estimated (Hodda 2022). However, a large number of species remain undescribed. Phylogenetic analyses based on molecular sequences, especially the small subunit rRNA gene (SSU), combined with morphological characters has updated the classification of Nematoda (De Ley and Blaxter 2002, 2004), revealed relationships among nematode groups (Holterman et al. 2006; Meldal et al. 2007), and elucidated the transition between marine, freshwater, and terrestrial different habitats (Holterman et al. 2008, 2019). However, due to the scarcity of nematode sequences, especially those of marine nematodes, relationships among nematodes are far from understood.

Species of the order Chromadorida Chitwood, 1993 are mainly marine species, and freshwater and limnetic–terrestrial species are also present among them. The Chromadorida are characterized by the combined characters of a punctate cuticle and a female reproductive system with reflexed ovaries (Lorenzen 1981), which distinguish this order from other nematode orders. Lorenzen (1994) summarized the superfamily Chromadoroidea (corresponding to Chromadorida) systematically and gave a phylogenetic analysis of this group based on morphological features. Tchesunov (2014) reviewed the six families of Chromadorida and provided diagnoses of the families and genera; he listed Chromadoridae with five subfamilies and 38 genera, Cyatholaimidae with four subfamilies and 21 genera, Achromadoridae with a single genus (*Achromadora*), Ethmolaimidae with three genera, Neotonchidae with six genera, and Selachinematidae with two subfamilies and 11 genera. Venekey et al. (2019) reviewed the family Chromadoridae, provided polytomous identification keys for subfamilies and genera, listed valid species of each genus, and provided a phylogenetic analysis based SSU rRNA gene sequences of 11 genera and large subunit (LSU) rRNA gene sequences of eight genera. Phylogenetic relationships within Chromadorida based on full-length sequences of the SSU rRNA gene has been analyzed for 4 families by Holterman et al. (2008) and six families (22 genera) by Leduc et al. (2017). As more Chromadorida SSU rRNA gene sequences have been deposited in the GenBank, an updated and more detailed phylogenetic analysis is needed.

Until now, 29 species of *Chromadorina* Filipjev, 1918 have been described, among which 25 species are marine and four are limnetic (*C.astacicola* W. Schneider, 1932, *C.bercziki* Andrássy, 1962, *C.bioculata* (Schultze, 1857) Wieser, 1954, and *C.viridis* (Linstow, 1876) Wieser, 1954). A new species, *C.communis* sp. nov., is described here and a molecular phylogeny based on SSU rRNA gene sequences within Chromadora is analyzed. The new species was found during a study of the diversity of free-living marine nematodes of Changdao Island. This is the first new species of *Chromadorina* from the China Sea.

## ﻿Materials and methods

### ﻿Sample collection and nematode identification

**Figure 1.**
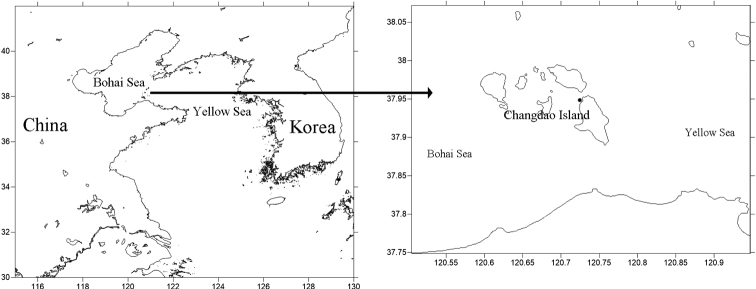
Sampling site (dot) on Changdao Island.

From Changdao Island in June 2022, undisturbed samples were collected from rock surfaces with *Ulvalactuca* and *U.prolifera* (Fig. 1). Samples used for morphological analysis were fixed with 10% formalin in seawater, and samples used for molecular analysis were fixed with 95% alcohol. In the laboratory, sediment samples fixed with formalin were washed through two sieves (mesh sizes 500 μm and 45 μm) to separate meiofauna from macrofauna larger than 500 μm. Meiofauna were transferred into a grid-lined Petri dish and sorted under a stereoscopic microscope. Nematodes were transferred into a mixture of ethanol (50%) and glycerin (ratio 1:9 by volume), the ethanol was allowed to slowly evaporate (McIntyre and Warwick 1984), and the specimens were mounted in glycerin on permanent slides. Descriptions were made using an Axiscope-5 differential interference contrast microscope (Zeiss, Germany). Line drawings were made with the aid of iPad (Apple, USA), and photographs were taken with the aid of ZEN software (Zeiss).

### ﻿DNA extraction, PCR amplification, and phylogenetic analysis

Samples used for molecular analysis were washed and separated as with formalin-fixed samples. Species of *C.communis* sp. nov. were selected and confirmed on the temporary slides based mainly on buccal cavity, spicules, and numbers of precloacal supplements. Genomic DNA of seven nematode specimens were extracted with DNeasy Blood & Tissue kit (Qiagen, Germany) and used as templates of nearly full-length SSU rRNA gene amplification with two sets of primers, 1096F (5’ – GGT AAT TCT GGA GCT AAT AC – 3’) / 1912R (5’ – TTT ACG GTC AGA ACT AGG G – 3’) and 1813F (5’ – ctg cgt gag agg tga aat – 3’) / 2646R (5’ – gct acc ttg tta cga ctt tt – 3’) (Holterman et al. 2006). PCR was conducted as described by Zhao et al. (2015). The PCR product was sequenced by Genewiz (China). The sequences were assembled in Genious v. 6.1.2. The new SSU sequence was deposited in GenBank under accession number OP680597. Near full-length SSU rRNA sequences of Chromadorida and Desmodorida (genus *Prodesmodora* Micoletzky, 1923) were retrieved from GenBank with BLAST; other sequences in GenBank from Chromadorida and longer than 600 bp were also used in the phylogenetic analysis.

Sequences were aligned with the Clustal W algorithm, and the final alignment consisted of 76 sequences from 34 genera. Substitution models of (GTR (general time-reversible) + G (gamma distribution) + I (proportion of invariable sites)) were selected as the best-fit model for SSU alignments. A ML analysis was performed with Mega X with 1000 bootstrap replicates. A BI analysis was constructed with CIPRES (http://www.phylo.org/) and MrBayes on XSEDE v. 3.2.7a was used; the trees were run with chain length of 10,000,000, burn-in frac = 0.25, and the analysis was rooted with *Enoplolaimus* sp. (accession number KR265034). The topology of the resulting tree was viewed in FigTree v. 1.4.3 and edited with PowerPoint.

## ﻿Results and discussion

### ﻿Taxonomy

**Order Chromadorida Chitwood, 1933**

**Family Chromadoridae Filipjev, 1917**

#### ﻿Genus *Chromadorina* Filipjev, 1918

##### ﻿ Chromadorina communis sp. nov.

Taxon classificationAnimaliaChromadoridaChromadoridae

6F69C689-BFA2-5228-820E-3D855EA57960

https://zoobank.org/63F40161-62AA-4836-8F27-9AAF48185A28

**Figure 2.**
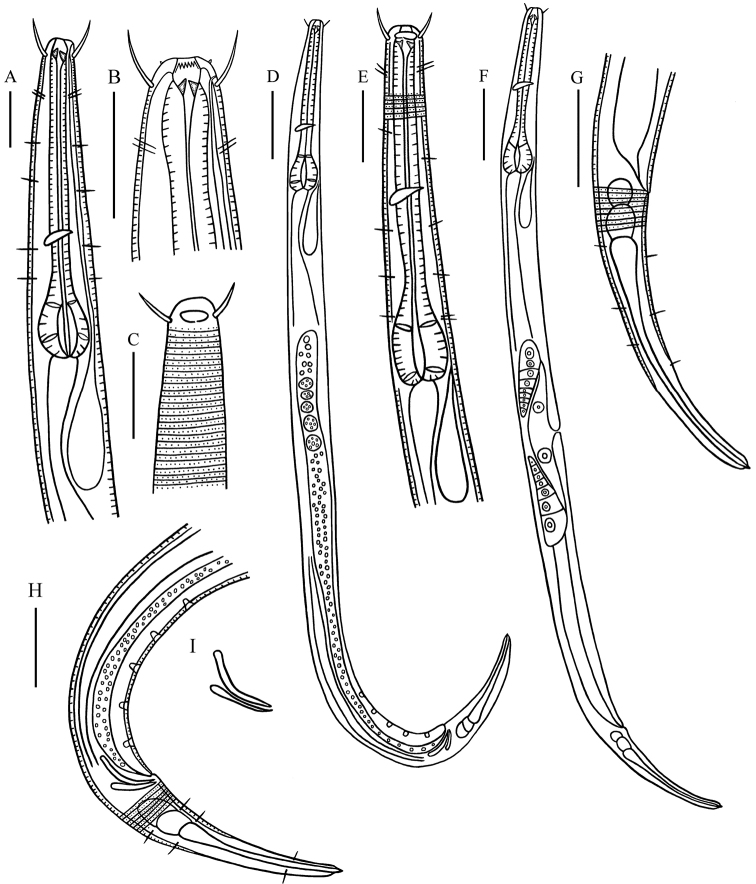
*Chromadorinacommunis* sp. nov. **A** lateral view of male anterior region showing buccal cavity and pharyngeal region (holotype) **B** lateral view of male anterior region showing buccal cavity (holotype) **C** lateral view of male anterior region showing cuticle and amphids (holotype) **D** lateral view of male whole body (holotype) **E** lateral view of female anterior body showing buccal cavity and pharyngeal region (22YTCD6-2-1) **F** lateral view of female entire body showing vulva (22YTCD6-2-1) **G** lateral view of female posterior body (22YTCD6-2-1) **H** lateral view of male posterior body, showing precloacal supplements and tail (holotype) **I** lateral view of spicules and gubernaculums. Scale bars: 20 µm (**A–C, E**); 50 µm (**D, F**); 30 µm (**G, H**).

**Figure 3.**
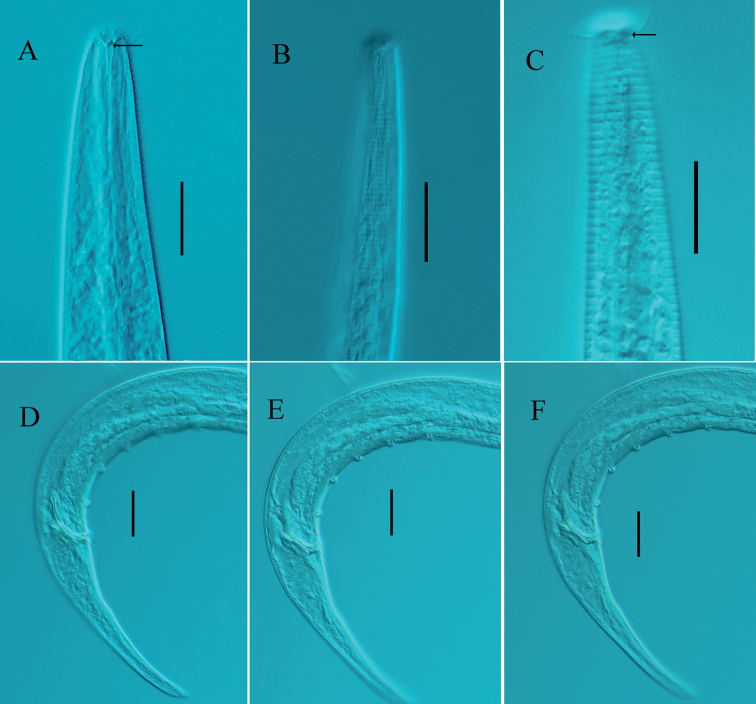
*Chromadorinacommunis* sp. nov. **A** lateral view of male anterior region showing buccal cavity and teeth (arrow) (holotype) **B** lateral view of male anterior region showing cuticle (holotype) **C** lateral view of female anterior region showing amphids (arrow) and cephalic setae (22YTCD6-2-1) **D–F** lateral view of male posterior body showing spicules, precloacal supplements, and gubernaculum (holotype). Scale bars: 20 µm.

Figs 2 , 3 , Table 1

###### Diagnosis.

*Chromadorinacommunis* sp. nov. is characterized by its medium-sized body, finely striate cuticle with punctuations, buccal cavity with three equally sized teeth, absent ocelli, four cephalic setae 6–8 μm long, oval amphidial fovea level with cephalic setae, curved spicules with tapered distal end, boat-shaped gubernaculums, five or six cup-shaped precloacal supplements, and a conical tail with a short spinneret.

###### Material examined.

Four males and two females were measured and studied. ***Holotype***: m#1 on slide 22YTCD6-2-17; ***paratypes***: m#2 on 22YTCD6-2-15, m#3 on 22YTCD6-2-18, m#4 on 22YTCD6-2-11, f#1 on 22YTCD6-2-1, and f#2 on 22YTCD6-2-18. Type specimens were deposited in the Institute of Oceanology, Chinese Academy of Sciences, Qingdao.

###### Measurements.

Detailed measurements information of individual specimens are shown in the Table 1.

**Table 1.** Measurements of *Chromadorinacommunis* sp. nov. (in µm except for ratios).

Characters	Holotype	Paratypes	Paratypes
	male	males (*n* = 3)	females (*n* = 2)
Total body length	711	741.0 ± 73.6 (696–851)	753.5 ± 47.4 (720, 787)
Maximum body diameter	32	27.8 ± 2.9 (26–32)	31.5 ± 0.7 (31, 32)
Head diameter	11	10.5 ± 0.6 (10–11)	10.5 ± 0.7 (10, 11)
Length of cephalic setae	8	6.5 ± 1.0 (6–8)	6.5 ± 0.7 (6, 7)
Diameter of amphidial fovea	5	4.5 ± 0.6 (4–5)	5.0 ± 0.0 (5, 5)
Amphidial fovea*	2	2.5 ± 0.6 (2–3)	2.0 ± 0.0 (2, 2)
Nerve ring*	71	71.5 ± 4.7 (67–78)	72.0 ± 0.0 (72, 72)
Body diameter at nerve ring	24	21.8 ± 1.5 (21–24)	21.5 ± 0.7 (21, 22)
Pharynx length	119	122.5 ± 5.1 (118–129)	125.5 ± 4.9 (122, 129)
Pharynx bulb length	26	24.8 ± 1.5 (23–26)	27.5 ± 0.7 (27, 28)
Body diameter at the base of pharynx	28	24.5 ± 2.4 (23–28)	25.5 ± 0.7 (25, 26)
Anal body diameter	22	22.0 ± 1.4 (21–24)	17.5 ± 2.1 (16, 19)
Spicules length along arc	28	27.5 ± 0.6 (27–28)	-
Gubernaculum length	17	17.5 ± 1.3 (16–19)	-
Vulva*	-	-	360.0 ± 11.3 (352, 368)
Body diameter at vulva	-	-	31.5 ± 0.7 (31, 32)
V%	-	-	47.9 ± 1.5 (46.8, 48.9)
Precloacal supplements	5	5.5 ± 0.6 (5–6)	-
Tail length	89	92.0 ± 5.7 (87–100)	115.5 ± 12.0 (107, 124)
a	22.2	26.9 ± 3.8 (22.2–27.1)	23.9 ± 1.0 (23.2, 24.6)
b	6.0	6.1 ± 0.4 (5.6–6.6)	6.0 ± 0.1 (5.9, 6.1)
c	8.0	8.1 ± 0.3 (7.7–8.5)	6.5 ± 0.3 (6.3, 6.7)
c’	4.0	4.2 ± 0.2 (4.0–4.4)	6.6 ± 0.1 (6.5, 6.7)

Abbreviations are as follows: a = body length/maximum body diameter; b = body length/pharynx length; c = body length/tail length; c’ = tail length/anal body diameter; V% = position of vulva from anterior body end expressed as a percentage of total body length. *Distance from anterior body end.

###### Description.

**Males.** Body cylindrical and medium-sized. Inner and outer cephalic setae inconspicuous. Four cephalic setae 6–8 μm in length (0.55–0.73 head diameter long), two pairs of sublateral cervical setae (4–5 μm long) present. Somatic setae (3–4 μm long) scarcely present in pharynx and on caudal region. Cuticle faintly striate, with homogeneous punctuations without longitudinal differentiation. Ocelli absent. Amphidial fovea oval, level with cephalic setae, in some specimens difficult to observe. Buccal cavity slightly cuticularized and funnel-shaped, with three equally sized, solid teeth. Pharynx cylindrical, with posterior end widened into obvious bulb (20.1–21.8% of pharynx length). Nerve ring slightly posterior to middle pharynx region (54–63% of pharynx length). Secretory–excretory system pore on anterior end of body (3–4 μm from anterior end).

Reproductive system monorchic, with anterior testis outstretched, located to right of intestine. Spicules paired, curved, 1.2–1.3 cloacal body diameters long; proximal end slightly cephalate and distal end tapered. Gubernaculum simple, boat-shaped, parallel to the distal end of spicules. Five or six cup-shaped precloacal supplements, 12 μm from cloacal opening and 12–16 μm apart. Tail conical, with short spinneret, with three caudal glands in line.

**Females.** Similar to males in most characteristics. Reproductive system didelphic and amphidelphic, with reflexed ovaries. Anterior ovary to right of intestine and posterior ovary to left of intestine. Vulva slightly anterior to mid-body. Vagina short and sclerotized.

###### Type locality and habitat.

Changdao Island, Shandong Province, China, 37°57'N, 120°43'E, at the confluence of the Yellow and the Bohai seas. Salinity 28.1‰ ± 0.36.

###### Distribution.

Occurred on rock surfaces with *Ulvalactuca* and *U.prolifera*.

###### Etymology.

Latin, *communis*, “common”.

###### Differential diagnosis.

*Chromadorinacommunis* sp. nov. is similar to the cultured species *C.hirommi* Kito & Nakamura, 2001 in body length, length of cephalic setae, and numbers of precloacal supplements, but it differs in tooth shape (three equally sized teeth vs dorsal tooth large and subventral teeth small), absence of ocelli (brownish pigment present in *C.hirommi*), position of excretory pore (0.3 head diameter from anterior body end vs 1.8 head diameter from anterior body end), spicule length and shape (27–28 μm, distal end tapered vs 22–25 μm, distal end blunt and bifurcate), and gubernaculum shape (slightly cuticularized and boat-shaped vs well cuticularized with wavy dorsal fringe, distal parts of lateral pieces distinct and directed ventrad).

Eleven sequences of *Chromadorina* are included in the phylogenetic analysis, but only three species (*C.bioculata*, *C.germanica*, and *C.communis* sp. nov.) are identified to species, and *C.bioculata* was found in freshwater. *Chromadorinacommunis* sp. nov. shows a close relationship with *Chromadorina* sp. (KJ636255), but it differs by 3.1% (52 in 1678 bp, including three gaps). *Chromadorinacommunis* sp. nov. is supported as a new species by both phylogenetic and morphological analyses.

### ﻿Molecular phylogenetic relationships and analysis

**Figure 4.**
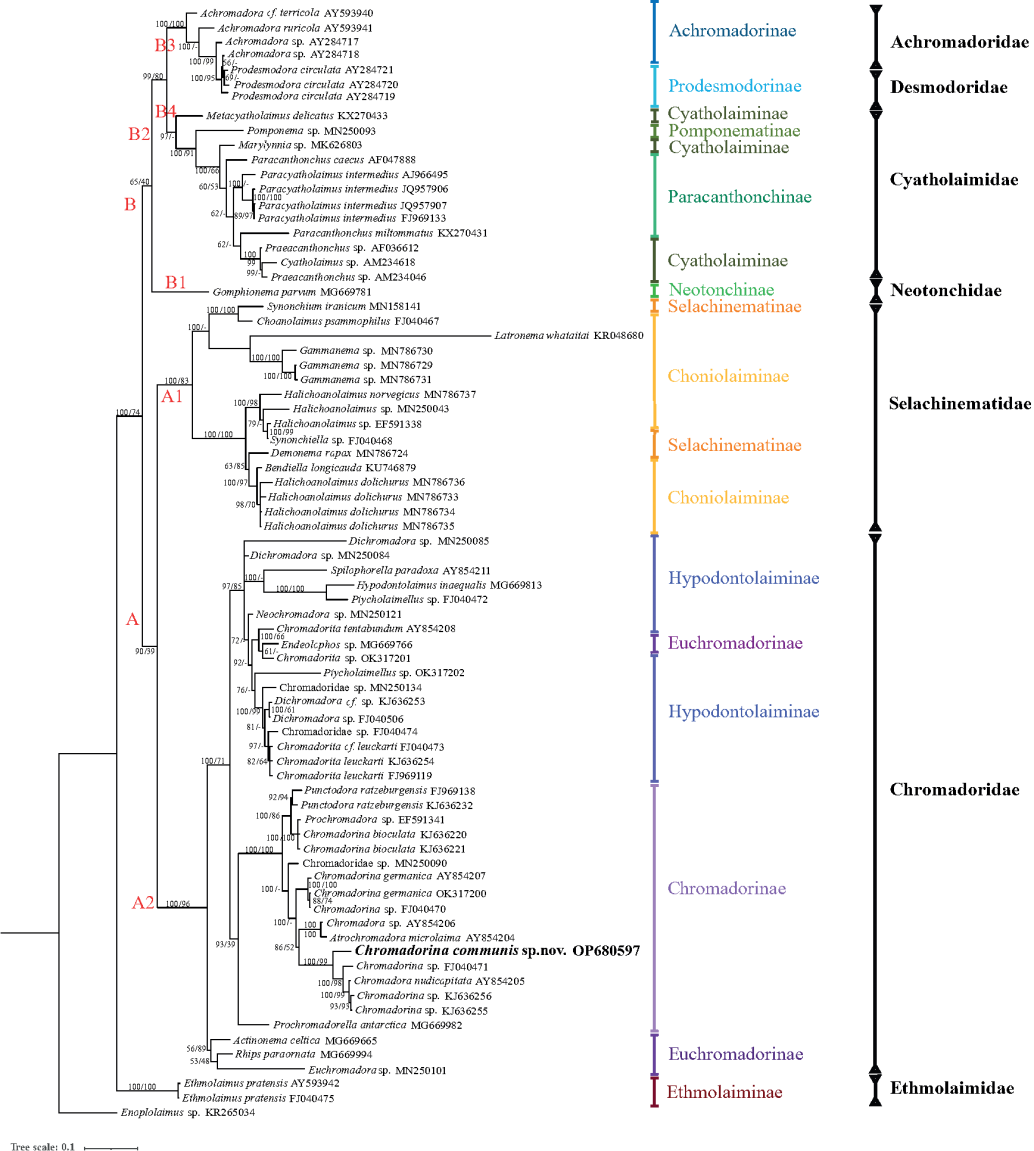
Bayesian inference tree of the order Chromadorida inferred from small subunit (SSU) sequences under the general time-reversible (GTR) + proportion of invariable sites (I) + gamma distribution (G) model. Posterior probability (left) and bootstrap values (right) are given on corresponding clades. The sequence obtained in this study is shown in bold. Subfamilies and families are listed on the right. Main clades are shown by red letters or letters with numbers. The scale stands for substitutions per site.

The ML topology obtained with the SSU rRNA gene sequence is in accordance with the BI topology, and only the BI tree is shown (Fig. 4). The main Chromadorida clade is split into two major clades: Clade A composed of Selachinematidae and Chromadoridae and Clade B composed of Cyatholaimidae, Achromadoridae, Neotonchidae, and *Prodesmodora* (100% posterior probability and 74% bootstrap value). Ethmolaimidae (with one species only, *Ethmolaimuspratensis*) splits early from the main Chromadorida clade. Six Chromadorida families constitute well-supported monophylectic clades.

In clade A1, nine genera of Selachinematidae form a well-supported monophyletic group (100% posterior probability and 83% bootstrap value), but Selachinematinae and Choniolaiminae are not shown as monophyletic clades at the subfamily level. In Clade A2, Chromadoridae forms a well-supported monophyletic group (100% posterior probability and 96% bootstrap value). In the subfamily level, only Chromadorinae constitutes a monophyletic clade based on the BI analysis (93% posterior probability); however, it is weakly supported by the ML analysis (39% bootstrap value). Hypodontolaiminae and the genus *Endeolophos* (Euchromadorinae) constituted a monophyletic clade (97% posterior probability and 85% bootstrap value). *Endeolophos* shows a close relationship with the genus *Chromadorita*, which was highly supported by the BI analysis (100% posterior possibility), and it clustered with Hypodontolaiminae. The morphological characters of *Endeolophos*–cuticle homogeneous, amphidial fovea at the level of the cephalic setae, and gubernaculum without telamons–conform to the subfamily Hypodontolaiminae, while other characters–outer labial sensillar setiform, cuticular with complex structure, pharyngeal bulb absent, and precloacal supplements absent–conform to the Euchromadorinae (Tchesunov 2014). We prefer to keep *Endeolophos* in Euchromadorinae despite the molecular evidence.

*Chromadora* shows a close relationship with *Chromadorina* in clade A2, and Venekey et al. (2019) also noted this and explained this relationship as a result of a misidentification; there are great morphological similarities between *Chromadora* and *Chromadorina* (Venekey et al. 2019).

In clade B, *Gomphionemaparvum* (clade B1) of family Neotonchidae split early, but it is weakly supported (65% posterior probability and 40% bootstrap value). The position of Neotonchidae has been uncertain for a long time. Platt (1982) united Neotonchinae and Ethmolaiminae into Ethmolaimidae with the holapomorphy of “cup-shaped precloacal supplements with an external articulated flange”, but this character was doubted by Lorenzen (1994), as it also occurs in *Dichromadora*. Tchesunov (2014) also considered the validity of the family Neotonchidae due to its mixed characters of Chromadoridae, Cyatholaimidae, and Microlaimidae. According to our BI and ML analyses, Neotonchinae (clade B1) is a sister taxon to Achromadoridae (clade B2), *Prodesmodora* (clade B3), and Cyatholaimidae (clade B4), but there is no close relationship with Ethmolaiminae. This is in accordance with the topology tree by Leduc et al. (2017), but it is contrary to Bezerra et al.’s (2013) results based on morphological characters. We prefer to retain Neotonchidae until more molecular evidence is available.

In clade B3, two freshwater genera, *Achromadora* and *Prodesmodora*, form a highly supported clade (100% posterior probability and 100% bootstrap value). Lorenzen (1981, 1994) concluded that *Achromadora* is a holophylectic based on the the position of the ovary and prevailing parthenogenetic reproduction, and holapomorphy was presented in *Prodesmodora*. Taxonomic position of *Prodesmodora* should be reconsidered.

## ﻿Conclusions

*Chromadorinacommunis* sp. nov. is described based on morphological characteristics and distinguished from allied species by the absence of ocelli, a buccal cavity with three equally sized, solid teeth, curved spicules with a tapered distal end, simple and boat-shaped gubernaculums, and a conical tail with a very short spinneret. A phylogenetic analysis also supports the validity of the new species. With the description of *C.communis* sp. nov., 30 species of *Chromadorina* have been identified.

The phylogenetic analysis of Chromadorida show six families clustered into a monophyletic clade, and this conforms to the morphological taxonomy at the family level. The genus *Endeolophos* should be kept in Euchromadorinae based on morphological characters, and the position of family Neotonchidae should be considered as valid until further data are available.

## Supplementary Material

XML Treatment for Chromadorina communis
